# 15 Smartphone Apps for Older Adults to Use While in Isolation During the COVID-19 Pandemic

**DOI:** 10.5811/westjem.2020.4.47372

**Published:** 2020-04-14

**Authors:** Swechya Banskota, Margaret Healy, Elizabeth M. Goldberg

**Affiliations:** * The Warren Alpert Medical School of Brown University, Providence, Rhode Island; † Johnson & Wales University, Providence, Rhode Island; ‡ The Warren Alpert Medical School of Brown University, Department of Emergency Medicine, Providence, Rhode Island; § Brown University School of Public Health, Department of Health Services, Practice and Policy, Providence, Rhode Island

## Abstract

The maintenance of well-being, healthcare, and social connection is crucial for older adults (OA) and has become a topic of debate as much of the world faces lockdown during the coronavirus disease 2019 (COVID-19) pandemic. OAs have been advised to isolate themselves because they are at higher risk for developing serious complications from severe acute respiratory syndrome coronavirus. Additionally, nursing homes and assisted-living facilities across the country have closed their doors to visitors to protect their residents. Mobile technology such as applications (apps) could provide a valuable tool to help families stay connected, and to help OAs maintain mobility and link them to resources that encourage physical and mental well-being. Apps could address cognitive, visual, and hearing impairments. Our objective was to narratively summarize 15 apps that address physical and cognitive limitations and have the potential to improve OAs’ quality of life, especially during social distancing or self-quarantine.

## INTRODUCTION

In January 2020, the first case of coronavirus disease 2019 (COVID-19) was identified in the United States. Shortly thereafter, visitation restrictions and guidance to reduce contact with older adults (OA), ≥ age 65, were put in place at many facilities caring for OAs with the aim to protect them from infection.^1^–^3^ According to the World Health Organization, the case fatality rate for COVID-19 in older adults in China 80 years and older was 21.9% compared to 1.4% for people of all ages with no underlying health conditions.^4^ However, as many state and civic leaders are now debating lockdowns many OAs may lack the assistance they need at home or in facilities to meet their daily needs. Self-imposed and/or institution-imposed social distancing could make OAs feel isolated, anxious, and sorrowful over their loss of independence and connections to friends and family.

OAs ≥ age 65 are increasingly using mobile technologies (MT) for healthcare purposes.^5^ MTs such as applications (app) could help OAs stay connected to friends and family, remain active, and access resources to address their daily nutritional, physical, and mental health needs. Therefore, MTs and apps can be useful to OAs by limiting their need to leave their residences, and risk exposure to COVID-19 by helping them remain in contact with loved ones, have access to meal delivery services, electronic access to healthcare providers to see to their chronic health conditions, and physical and cognitive impairment aids.

MTs can address loneliness and isolation, which have been associated with higher risks of depression and cardiovascular risk factors in OAs.^6^,^7^ Digital technology can enhance well-being and improve social connectedness by improving social support and engagement in activities.^8^,^9^ Although the positive effect of the use of information and communication technologies on social connectedness and social support seems to be short term, lasting less than six months,^9^ these tools could provide help during the critical first months of the COVID-19 pandemic to protect this population from the risks of loneliness and social isolation. Accessible to those with smartphones and internet connection, various apps may be useful tools for OAs so that they do not have to battle social distancing in isolation.

Nine in ten OAs who own MTs reported they use them to initiate communication through text messages or emails, obtain traffic information and news, and purchase apps.^10^ Sixty-nine percent of smartphone-using participants had downloaded or purchased apps before.^10^ Only 18% felt confident about data safety, highlighting that privacy and security are major concerns for them.^10^–^11^ Although OAs own MT and use apps, apps designed to enhance physical and mental health are not being used and/or recommended, nor have OAs been well educated in the relative safety and security of these apps.^12^ Additionally, previous research has shown that even though OA technology ownership rates are high, with four in ten older adults owning smartphones, the usability rates are low,^5^ which implies that OAs may need some guidance in both choosing and using apps that could benefit them. This article is intended to provide guidance to clinicians and family members seeking to help their older patients or loved ones during the COVID-19 pandemic and in other situations where isolation occurs.

## METHODS

In this narrative review of apps for OAs, we aimed to find apps available to OAs on the Apple Store that could potentially facilitate health during times of social distancing and/or self-quarantines. These apps were curated by a research team that included an emergency medicine attending and physician scientist in geriatrics and digital health, a medical student, a graduate student in biotechnology, and others. The apps are categorized by common healthcare needs within the OA population addressed by the following categories: 1) social networking; 2) medical, with subcategories a) telemedicine and b) prescription management; 3) health and fitness; 4) food and drink; and 5) visual and hearing impairment. App categories were determined based on app categories already in place on the Apple Store, with the exception of a category to address the specific needs of OAs with visual and hearing impairment, for which we did a custom search using the terms “blind” and “deaf” Details about the app developer, cost (both to download and for services included in the app), function, ratings and reviews, and user experience (in the form of anecdotes) were searched and summarized. All app rating and review data was last updated to this article on March 18, 2020.

### Inclusion and Exclusion Criteria

**Figure 1:**
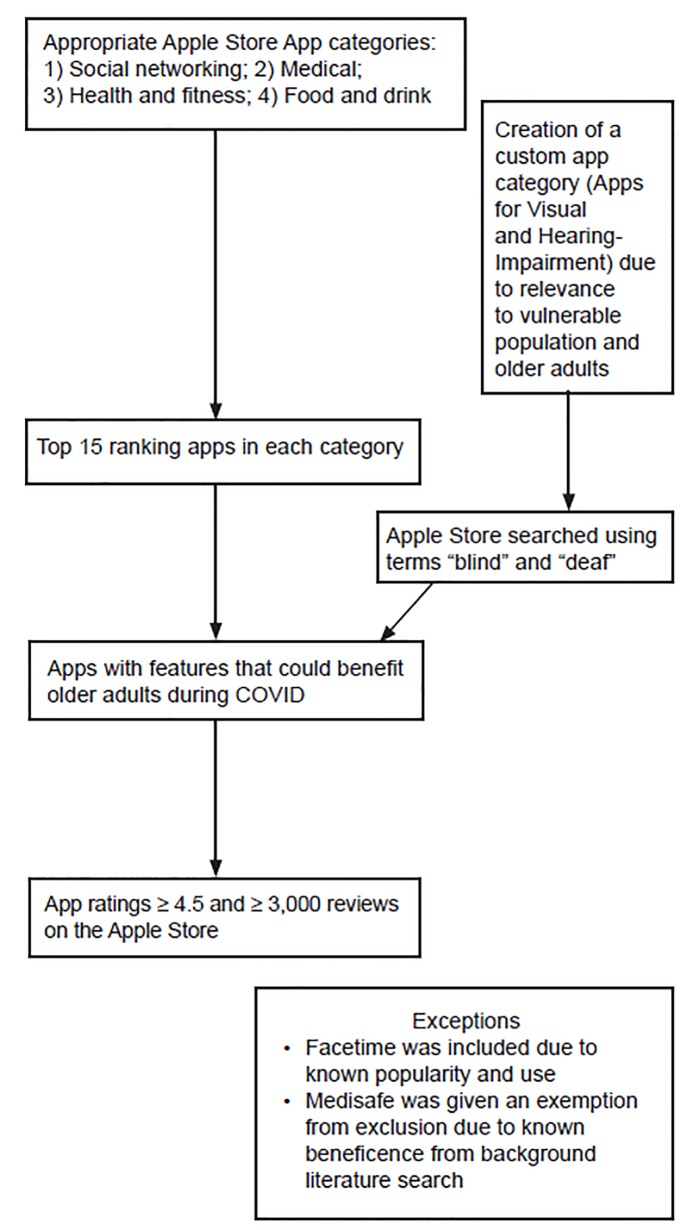
Inclusion and exclusion criteria for 15 smartphone apps for older adults to use during the coronavirus disease 2019 pandemic. *COVID*, coronavirus disease.

In the final list of 15 apps, we aimed to include those that are either designed to target the OA population or have features that could benefit OAs during pandemics and outbreaks when social isolation and/or self-quarantine is encouraged. Apps with broad acceptability were given priority. Hence, apps needed a rating of 4.5 or higher and at least 3000 reviews on the Apple Store. Exceptions were given for apps with broad appeal and applicability to the objective, such as FaceTime, Medisafe, and apps that assist people with vision and hearing impairment, as shown in Figure 1. Apps were further screened based on function and then ranking. Users’ experiences of the app were given consideration during the selection; hence, recent customer reviews that demonstrated that the app was a valuable product for an OA were selected and summarized as anecdotes. We conducted a literature review using PubMed and Google Scholar on the topic, but as many apps are not rigorously tested for usability and efficacy in the OA population, this selection was mainly based on expert review.

**Population Health Research Capsule**

What do we already know about this issue?

Older adults (OA) need support to address daily needs and maintain their mental and physical health as they practice social isolation during the pandemic.

What was the research question?

Are there smartphone apps that could potentially address OAs’ health and daily needs during the COVID-19 pandemic?

What was the major finding of the study?

We found 15 inexpensive and accessible smartphone apps that could support OAs during the pandemic.

How does this improve population health?

These apps enable OAs to stay connected and maintain independence and health while practicing social isolation.

## RESULTS

We list several apps that assist OAs with daily needs. These are summarized by cost and intended use in Table 1. User ratings and reviews, in the form of anecdotes, are provided in Table 2.

## DISCUSSION

Many apps are available to help OAs navigate isolation during the COVID-19 pandemic. While not all of the apps on our list are marketed specifically to OAs, we include apps with broad acceptability and positive user experience to ensure a list that helps access healthcare, maintain mental and physical health, and meets OAs’ various social and functional needs during social distancing during the COVID-19 outbreak. These apps could also provide OAs fearing loss of independence a sense of purpose and control over their life and health.

### Social Networking Apps (FaceTime and Skype)

Social isolation and self-quarantine, whether it is self-imposed, legally and/or institutionally mandated, can lead to negative impacts on an OA’s mental and physical well-being.^13^ The impact of social isolation on health could be as harmful as traditional risk factors such as high blood pressure, smoking, and obesity.^14^ Even before COVID-19, 28% (13.8 million) of OAs were living alone.^15^ Social isolation has been linked to physical and cognitive conditions including heart disease, high blood pressure, anxiety, depression, Alzheimer’s disease, and a weakened immune system.^15^ Fortunately, MT could provide a solution to isolation by enhancing the connection with loved ones in a safe and easy way, through apps such as FaceTime and Skype. Although MT cannot replace face-to-face interaction, it can still provide ease for those who feel a loss of connection.

OAs who use video chat apps, including FaceTime and Skype, are estimated to decrease their symptoms of depression by half.^16^ In a survey of 1400 OAs, those who use video chats were found to have lowered probability of depression symptoms, whereas depression rates among OAs who use instant messaging and social media networks were similar to OAs who do not use any communication technology.^16^ Skype is the oldest video chat app that offers the widest device support, including for Android, iOS, Windows Phone, and Blackberry. It can run on desktop software including Windows PC and Apple’s MacBook.^17^ Nursing homes and OA living residences frequently use Skype to connect OA residents to their loved ones, even though the app takes some explanation to learn the software so users can fully understand how to use it.^6^ Additionally, per recent policy changes by the US Department of Health and Human Services (HHS) Office for Civil Rights (OCR), Medicare beneficiaries may have improved access to their medical providers through FaceTime and Skype by approving reimbursement at the same rate for an in-person as a telemedicine visit.^18^

### Food & Drink Apps (DoorDash & Instacart)

Food and drink apps on the Apple Store can be a solution for vulnerable populations as users have access to same-day delivery services such as DoorDash and Instacart, allowing them to remain in their homes and maintain social distance. DoorDash has implemented “No-Contact Delivery Options” as a response to COVID-19. The app allows users to fill out personalized delivery instructions, requesting drivers to leave orders outside to avoid person-to-person contact.^19^ Users have the ability to text pictures and/or descriptions to where drivers should place their order, which may be easier for some than typing due to the loss of dexterity with aging. Due to the closure of many restaurants, individuals should verify that a restaurant is open before placing an order. Instacart, a grocery delivery service, has seen a surge in demand for the month of March 2020 due to COVID-19, especially in states with an increased number of cases, and also promises drop-off delivery that minimizes contact.^20^ These apps can cater to the OA population by giving them the option to stay home or providing families with the option to order food for their older loved ones rather than deliver it on their own, if they themselves are in quarantine.

### Medical Apps: Telemedicine Apps (Doctor on Demand, Teladoc, and K Health: Primary Care)

As a COVID-19 response, hospitals and clinics across the country have started to defer elective appointments and surgeries.^21^,^22^ OAs may benefit from this restriction due to reduced exposure to the virus, but many have chronic health conditions that need to be addressed. Telemedicine may provide a temporary solution for these needs. The Centers for Medicare & Medicaid Services’ recent expansion of Medicare coverage for telehealth services to its beneficiaries provides an alternative for in-person medical care, and the waiver of Medicare’s cost-sharing requirements for COVID-19 will improve access to care.^18^,^23^ CMS requires services provided via telehealth to be used for patients with an established relationship with the provider (but will not conduct audits to ensure this), and that “providers must use an interactive audio and video telecommunications system that permits real-time communication.”^18^

Medical apps that provide telehealth could facilitate care “early during the course of an acute problem or chronic disease exacerbation,” and provide healthcare access to those patients who have never had a prior correspondence with a provider.^24^,^25^ These resources could be valuable to uninsured and undocumented OAs in the US.^26^,^27^ These platforms may also be viewed as an extra resource that provide patients, especially those living in medically underserved areas, where access to care is limited.

These platforms can connect patients to remote physicians during emergency closures and during times of increased demand for medical services.^28^ For example, during Hurricanes Harvey and Irma, Doctor on Demand offered visits for chronic conditions, advice, counseling, and refills, and back and joint concerns.^29^ Doctor on Demand, Teladoc, and K Health: Primary Care are options available on the Apple Store that provide access to licensed physicians for non-emergency medical problems and are Health Insurance Portability and Accountability Act of 1996 (HIPAA) compliant.^30^–^32^ Doctor on Demand and Teladoc are considered leaders in telemedicine, and are covered by many insurances including UnitedHealthcare, Aetna, Cigna, and some state Medicaid programs, although coverage may be different, and different insurances have different preferred telehealth destinations.^30^,^32^–^35^ It is also important to note that many states have made changes to their telemedicine license policies due to COVID-19.^23^,^36^

The fact that our healthcare system was not equipped to provide telehealth on a mass scale for an outbreak is demonstrated by the waiver of penalties for HIPAA violation for using “everyday communication technologies such as FaceTime and Skype” to provide medical care during the COVID-19 emergency.^18^ In contrast, smartphone apps we have listed that provide telehealth services ensure HIPAA-compliant services, which may be preferred by some patients with privacy concerns.

Telemedicine has not always been embraced as a viable solution for patients.^37^ Providers in these platforms do not have access to key information from physical examination and diagnostic testing; in addition, they lack access to care coordination and insight gained from longitudinal care.^24^,^37^ However, telemedicine may be the only viable solution during COVID-19, and many experts predict OAs could benefit long term from the improved access to care these platforms provide. Telehealth clinicians have experience working with limited exam and diagnostics tools and should acknowledge when an actual visit is necessary due to the acuity of the condition or the need for an in-person exam or procedure. Patients are generally satisfied with telehealth service use.^38^,^39^ Therefore, access to care during this time may contribute to reduction of anxiety and frustration, in addition to feelings of loneliness, in the OA population.

It is important to note that racial disparity is known to exist in telemedicine access, as well as that the majority of current telemedicine users are younger adults.^25^ Therefore, ensuring equity in telemedicine access is important during this crisis, along with special effort in introducing and orienting OAs from under-represented backgrounds.

### Medical Apps: Medication-related Apps (Medisafe & GoodRx)

In adults 60 years and older, more than 76% use two or more prescription drugs and 37% used five or more (called polypharmacy).^40^ Furthermore, per the Kaiser Family Foundation, “about one-fifth of older adults report[ed] not taking their prescribed medication as prescribed due to cost.”^41^ GoodRx provides discounts on medication, which could be particularly useful for OAs with a limited budget or high out-of-pocket costs due to being on multiple medications. According to an AARP survey, 32% of midlife adults provided regular financial support for basic necessities to their parents regularly in 2019, and more than a quarter of these adults reported that this caused them financial strain.^42^ Hence, GoodRx may be useful for adults financially supporting older parents, and for working Americans laid off due to business shutdowns.^43^

This is also a time when family members and caregivers who typically visit OAs and check on their medications are unable to do so because of social isolation and visitor restrictions at nursing homes and assisted living facilities. Medisafe could help OAs with trouble adhering to a medication regimen due to cognitive impairment or polypharmacy. Self-reported medication nonadherence is common in community-dwelling older adults especially in those with cardiovascular disease.^44^ Cardiovascular disease is a known risk factor for mortality among OAs who contract COVID-19.^45^

Medication nonadherence itself can be dangerous, as it contributes to more than 10% of hospital admissions in older adults, and is associated with increased incidence of heart failure.^46^,^47^ Hospital admissions may increase risk of exposure to COVID-19, and heart failure is associated with worse prognosis in OAs with COVID-19.^45^ Thus, OAs should be especially careful about medication adherence during this pandemic to protect health. In one study, participants using Medisafe had a small improvement in self-reported medication adherence.^48^ Therefore, Medisafe, along with its real-time missed medication alerts and frequent check-ins via phone calls by family members or healthcare providers, may help OAs stay in the path of medication adherence. In 2015, Medisafe announced a partnership with GoodRx to help lower medication costs.^49^ Medisafe along with GoodRx could help reduce barriers to medication adherence.

### Health and Fitness Apps (Calm, Headspace, Yoga: Down Dog, and MyFitnessPal)

OAs are prone to worrying about their health.^50^ Anxiety could be exacerbated during the COVID-19 crisis. Health anxiety has been found to be associated with more “distress, impairment, disability and health service utilization.”^51^ This finding underscores the importance of curating apps targeting health applications for OAs mental health. A study shows that OAs are “motivated to use digital technologies to improve their mental health.”^52^ In a study with participants aged 18–49, frequent use of Headspace for 30 days was associated with improvement in mental health, specifically depressive symptoms and resilience.^53^ In another study among college students, students who used Calm for eight weeks reported reduced stress.^54^ Although there has been no published research looking at the effectiveness of using applications such ase Calm and Headspace in OAs, these apps could be a useful tool to address anxiety.^55^

Social isolation and quarantine can decrease physical activity and promote sedentary behavior, which is problematic in a population that already spends 60% of awake time engaged in sedentary activities.^56^ Sedentary behavior is associated with disability in activities of daily living, development of metabolic syndrome, and an increased risk of all-cause mortality in the elderly.^57^ Long duration of sitting is negatively associated with femoral bone mineral density (FBMD) in women, whereas duration of light intensity physical activity is positively associated with FBMD.^58^ Physical activity intervention has been proven effective in improving physical activity behavior in healthy OAs, and most sequences of yoga are classified as a light-intensity physical activity.^59^,^60^ Some small studies also suggest that, in OAs, yoga may be superior to conventional physical-activity intervention.^61^

Suggesting healthy OAs to use an app such as Yoga: Down Dog could reduce the ill-effects of sedentary behaviors. Encouraging OA users to set a goal to pursue daily physical activity during social isolation and may serve as behavior intervention.^59^ Yoga could protect psychological health in this difficult time, and help with sleep quality.^62^,^63^ In a study in OAs, chair yoga participants had more improvement in anger, anxiety, depression, well-being, general self-efficacy, and self-efficacy for daily living than control and chair exercise participants.^62^

Chronic conditions common in OAs, such as hypertension and diabetes, can be controlled with exercise and good diet.^64^ MyFitnessPal, which provides a calorie counter and diet plan, could be a motivator for behavior change. MyFitnessPal is a behavior intervention that could provide benefit of well-being, but it requires self-efficacy.^59^,^65^ Limitations of MyFitnessPal include unreliable estimation of (micro-) nutrients ingestion and ineffectiveness in patients without goals and willingness to self-monitor calories.^66^–^69^ Therefore, although MyFitnessPal may be recommended to promote healthy behavior, OAs should not use MyFitnessPal by itself, and work in conjunction with a dietitian if possible.^70^

### Apps for Visual & Hearing-Impairment (Be My Eyes - Helping the Blind, and Glide - Live Video Messenger)

When asked about the vulnerable populations that have an increased risk of being affected by COVID-19, Dr. Lisa Cooper of Johns Hopkins reported that individuals with vision and hearing impairments are also vulnerable.^71^ As of 2016, an estimated four million OAs had vision disability.^72^ Vision impairments double the risk of falls, which one of four OAs experience, and are associated with morbidity and mortality.^73^ OAs with vision impairments who live alone and do not receive any caretaker service have to overcome greater challenges regarding activities of daily living and instrumental activities of daily living, which limits one’s quality of life and independence. Be My Eyes, the largest online support for the visually impaired, may be a useful resource to these OAs, especially at this time.^74^,^75^ Per Be My Eyes, over two million volunteers speaking over 180 languages have signed up on the app to assist those with impairments, increasing acceptance, socialization, and independence for this population.^75^,^76^ With the goal to help visually impaired individuals navigate through daily activities, volunteers have the ability to assist OAs who do not have support at home by keeping them safe, enabling users to have a sense of independence and support.^75^,^76^

An estimated one in three people between the ages 65–74 have difficulty hearing, with half of those older than 74 having difficulty hearing.^77^ OAs with hearing impairment have a greater chance of becoming depressed due to feeling frustrated and embarrassed about not understanding what is being said.^77^ Howard A. Rosenblum, chief executive officer of the National Association of the Deaf, stated that the US government must make information on COVID-19 accessible in American Sign Language (ASL), including information on how the virus affects education and employment access, among others.^78^ Glide - Live Video Messenger enables the ability to communicate to the hearing-impaired population through ASL and/or just videos. This may negate feelings of loneliness and depression during times of social distancing for COVID-19. Additionally, important information pertaining to disease characteristics, local and state business closures, financial updates, and other communications on COVID-19 could be shared to those with hearing impairments effectively and promptly using Glide.

## LIMITATIONS

**Figure 2:**
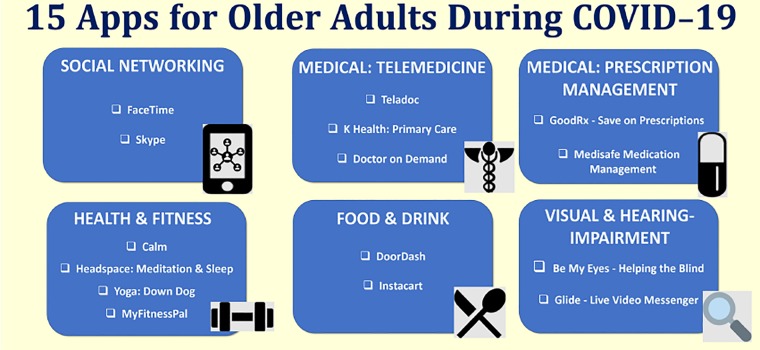
15 smartphone apps for older adults to use daily while in isolation during the coronavirus disease 2019 pandemic.

Our summary of the 15 apps, listed in Figure 2, was based on the functionality of apps on the Apple Store primarily using the “Top Charts” list and expert opinion. Rather than creating an exhaustive list, we focused on a brief list of apps that could be recommended to OAs during the COVID-19 pandemic. Apple Store is not accessible in all smartphones, and there is a far greater ownership rate of Android devices compared to iOS. However, except for FaceTime, the other apps on our list can also be found on Google Play Store, the Android app store. It is important to note that because app features may differ slightly on the two operating systems, user experience and ratings for the apps may vary between the two digital-distribution platforms.

Due to the limitations in our methodology, our 15 apps list does not address the barriers faced by older adults with hearing impairments but without experience using sign language. For these older adults, live captioning apps such as Ava, Otter.ai, and Microsoft Translator may be suggested. These apps can be downloaded on both iOS and Android devices. While Microsoft Translator is a completely free, Ava and Otter.ai is free for occasional use, which limits users to 5 hours/month and 600 minutes/month, respectively. Unlimited access can be purchased with a subscription to premium plans.

It is also critical to acknowledge that while digital health and MT use by OAs is increasing, few apps have been reviewed and tested for usability and efficacy in clinical trials among the OA population. In the future, additional research assessing the usability of these apps in the OA population using the Mobile App Rating Scale, or other usability models such as the technology acceptance model, should be conducted.^79^,^80^ However, many of the apps we have suggested fulfill an unmet need and could help OAs maintain physical and mental health, independence, address disabilities, and some financial security. Most importantly, they encourage and allow for a less imprisoning and isolating experience for OAs during this crisis.

## CONCLUSION

Apps are inexpensive and accessible, and research has shown that OAs can use smartphones when provided the necessary training.^81^ There is an increase in the use of smartphones in the aging population.^82^ Recommending these 15 apps, along with providing some training and guidance, to an OA could help decrease loneliness and maintain and/or improve the health and independence of OAs during the COVID-19 pandemic. While apps cannot substitute for all in-person care, they could supplement or substitute some in-person care.

## Figures and Tables

**Table 1:** Cost and function of 15 smartphone apps for older adults to use daily while in isolation during the coronavirus disease 2019 pandemic.

App Name	Developer	Cost	Function
Social Networking Apps			
FaceTime	Roberto Garcia, Apple Engineer	Free app built into Apple products upon purchase	May be used on any Apple products including iPhone, iPad, iPod Touch, and MacBook; enables phone and video call communication, either one-on-one or in groups between Apple product users.
Skype	Skype Technologies	Free to download the app and use features domestically. $2.99 monthly subscription for international use.	May be used on mobile devices and computers; allows for communication between Skype users via one-on-one or group phone or video calls.
Medical Apps: Telemedicine			
Teladoc	Teladoc	Free to download app. Expenses depend on the user’s health insurance (accept Medicaid, Medicare, and some commercial insurance). Per the Centers for Medicare & Medicaid Services (CMS) guidance, telehealth is covered at the same rate as in-person visits during the COVID-19 crisis.	Connects patients to a board-certified doctor 24/7 through phone visits. If needed, a prescription can be sent to the patient’s pharmacy.
K Health: Primary Care	K Health Inc.	Free to download app. Expenses depend on the user’s insurance. Per CMS guidance, telehealth is covered at the same rate as in-person visits during the COVID-19 crisis.	Provides digital primary care for patients and free risk assessments for COVID-19.
Doctor on Demand	Phil McGraw, Jay McGraw, Adam Jackson	Free to download app. App works with or without insurance and is available at reduced rates through many major health plans and large employers. The average cost of a video consultation copay with insurance is $24, and $99 flat rate fee without insurance. Per CMS guidance, telehealth is covered at the same rate as in-person visits during the COVID-19 crisis.	Provides face-to-face digital connection with a doctor, psychiatrist or psychologist through video on people’s iPhone or iPad; provides urgent care, behavioral health, preventive health, and chronic care management; provides services in many languages when appointment is scheduled.
Medical Apps: Prescription Management			
GoodRx-Save on Prescriptions	Trevor Bezdec	Free to download, but individuals may opt to purchase GoodRx Gold membership for $5.99/month per individual (and $9.99/month for up to six family members, including pets) for greater discount on prescriptions	An online app that finds prescription discounts and offers medication coupons.
Medisafe Medication Management (Medisafe)	Rotem Shor	Medisafe is free, but Medisafe premium monthly subscription is $4.99/month, and premium yearly subscription is $39.99/year.	Provides personalized medication reminders for each medication; provides vital drug interaction warnings; keeps users connected with caregivers through real-time missed medication alerts.
Health & Fitness Apps			
Calm	Michael Acton Smith and Alex Tew	Free to download and use limited version of app. Free 7-day trial of the premium version after which access costs $12.99/month, $59.99/year, and $299.99 for a lifetime subscription	App for mindfulness and meditation to lower stress and improve sleep.
Headspace: Meditation & Sleep	Headspace Inc.	Free to download, but costs $12.99 per month for access to the meditation sessions beyond the introductory ones. Alternatively, can cost $95 for an annual subscription.	Relaxation app with guided meditation and mindfulness techniques to lower stress and improve sleep.
Yoga: Down Dog	Yogi Buddhi Co.	Free to download app. Monthly subscription is $7.99/month, but until May 1st, users have access to all features due to COVID-19.	Allows users to practice yoga in their homes with over 60,000 configurations to create a new workout daily. Includes beginner and tailored OA classes.
MyFitnessPal	Under Armour Inc.	Free version available. Premium access costs $49.99 per year.	Online calorie counter and diet plan. Users can log exercise and step count.
Food & Drink Apps			
DoorDash-Food Delivery	DoorDash Inc.	Free to download but delivery and platform service fee and fee for meal; subscription fee of $9.99 a month available to receive unlimited, no-fee deliveries on orders of $15 or more (but subscription is currently only available in some areas).	Food delivery service. Allows users to order food from participating restaurants and cafes.
Instacart	Maplebear Inc.	Free to download, but fee for delivery service (can be paid per delivery basis, but delivery is free with monthly membership of $9.99 or annual membership of $99	Same-day grocery delivery that allows users to request specific items from grocery stores.
Apps for Visual & Hearing-Impairment			
Be My Eyes-Helping the Blind	Hans Jorgen	None	Connects blind and visually impaired people with sighted people who assist them with tasks.
Glide - Live Video Messenger	Glide	Free to download, and free for the first 90 days. A 3-month subscription costs $1.99, and a 1-year subscription costs $6.99.	Allows you to send “lightning-fast” video messages, enabling on-demand communication using sign language and visuals.

*COVID-19*, coronavirus disease 2019.

*OA*, older adult.

**Table 2:** Ratings and user reviews of 15 smartphone apps for older adults to use daily while in isolation during the Coronavirus Disease 2019 (COVID-19) pandemic.

App Name	Ratings	Anecdotes (“Review Title,” Year Review Was Posted)
Social Networking Apps		
FaceTime	Not available because it is a free app built into Apple products upon purchase.	Not available because it is a free app built into Apple products upon purchase.
Skype	4.5 stars; 41.5K ratings; #9 Social Networking	“Skype is easy and good to use in terms of functionality and interface. I use Skype phone to call international phones because the rate is very reasonable” (“Good and Easy To Use,” 2020).
Medical Apps: Telemedicine		
Teladoc	4.8 stars; 190K ratings; #4 in Medical	“This has become my go to for our family. We never have a long wait, the doctors are knowledgeable and we get our prescriptions right away. This service provides massive value” (“Always Reliable,” 2020).
K Health: Primary Care	4.8 stars; 8K ratings; #7 Medical	“All 3 of my kids were diagnosed with the flu. Discovered this app and wow it was a lifesaver. Spoke to the doctor and got my rx without having to leave the house” (“Great for Sick Mom,” 2020).
Doctor on Demand	4.9 stars; 48K ratings; #15 Medical	User did not have to leave home to get an antibiotic prescription at a local pharmacy, and reported, “What a fantastic service!” (“Amazing,” 2020).
Medical Apps: Prescription Management		
GoodRx- Save on Prescriptions	4.8 stars; 523K ratings; #2 in Medical	A patient was paying $50 dollars for a prescription until they switched to GoodRx. Now they are only paying $15 for the same medication (“Saving $$$,” 2020).
Medisafe Medication Management (Medisafe)	4.7 stars; 35K ratings; #113 in Medical	“My wife just came home from hospital with 3 medications from specialists and 1 medication from a primary doctor. I struggled to keep up until I started this app” (“Couldn’t do Without this App,” 2020).
Health & Fitness Apps		
Calm	4.8 stars; 748K ratings; #2 in Health & Fitness	“I struggle with anxiety anyway, and with a pandemic upon us, I’ve enjoyed using calm as a tool. I’ve used it during the day to deepen my meditation and yoga” (“Helpful,” 2020).
Headspace: Meditation & Sleep	4.9 stars; 623K ratings; #6 in Health & Fitness	“...Headspace is always my go-to for high quality soothing meditations. It has helped me calm down in the COVID-19 crisis, and Headspace is none other” (“Life-Changing,” 2020).
Yoga: Down Dog	4.9 stars; 95K ratings; #7 Health & Fitness	User states “this app helped improve my physical and mental well-being. I was able to start to learn more about yoga, build core strength, and flexibility” (“Great for Beginners,” 2020).
MyFitnessPal	4.7 stars; 946K ratings; #10 Health & Fitness	“I’ve tried many fitness apps in my life and My Fitness Pal has easily surpassed all others. It logs your food and nutritional facts so easily you can scan a barcode and it automatically logs it in your daily nutritional facts” (“Great App,” 2020).
Food & Drink Apps		
DoorDash- Food Delivery	4.8 stars; 5.8M ratings; #1 in Food & Drink	“I’m blind and use voiceOver. The app is easy to use and is fully accessible.” (“Paul’s Review,” 2020).
Instacart	4.8 stars; 735K ratings; #2 in Food & Drink	“...As a senior my daughters told me about Instacart. I love it. It’s easy for me to select my favorite brands and the delivery people have been so courteous. Long time, life time customer.” (“Long Time Customer,” 2020)
Apps for Visual & Hearing-Impairment		
Be My Eyes- Helping the Blind	4.7 stars; 4.3K ratings; no ranking	“I got a call to help someone out with their mail. After my call I had a huge smile on my face because it felt so good helping out...” (“Great app,” 2020).
Glide- Live Video Messenger	4.5 stars; 17K ratings; no ranking	“I use this app fairly regularly to communicate via ALS. It works great and I love the many features” (“Great for ASL,” 2020).
